# CROP – The Clinico-Radiologico-Ophthalmological Paradox in Multiple Sclerosis: Are Patterns of Retinal and MRI Changes Heterogeneous and Thus Not Predictable?

**DOI:** 10.1371/journal.pone.0142272

**Published:** 2015-11-13

**Authors:** Fahmy Aboulenein-Djamshidian, Martin Krššák, Nermin Serbecic, Helmut Rauschka, Sven Beutelspacher, Ivica Just Kukurová, Ladislav Valkovič, Adnan Khan, Daniela Prayer, Wolfgang Kristoferitsch

**Affiliations:** 1 Department of Neurology, SMZ-Ost Donauspital, A-1220 Langobardenstrasse 122, Vienna, Austria; 2 Karl Landsteiner Institute for Neuroimmunological and Neurodegenerative Disorders, A-1220 Langobardenstrasse 122, Vienna, Austria; 3 High Field MR Centre, Department of Biomedical Imaging and Image Guided Therapy, Medical University of Vienna, A-1090 Währingergürtel 18-20, Vienna, Austria; 4 Division of Endocrinology and Metabolism, Department of Internal Medicine III, Medical University of Vienna, Vienna, Austria; 5 Department of Ophthalmology, Medical University of Vienna, A-1090 Währingergürtel 18-20, Vienna, Austria; 6 Department of Ophthalmology, Medical Faculty Mannheim, University of Heidelberg, Theodor-Kutzer-Ufer 1-3, 68167 Mannheim, Germany; 7 Nuffield Department of Surgical Sciences, Division of Medical Sciences, University of Oxford, Oxford, United Kingdom; 8 Division of Neuroradiology and Musculo-Skeletal Radiology, Department of Biomedical Imaging and Image Guided Therapy, Medical University of Vienna, A-1090 Währinger Gürtel 18-20, Vienna, Austria; University of Jaén, SPAIN

## Abstract

**Background:**

To date, no direct scientific evidence has been found linking tissue changes in multiple sclerosis (MS) patients, such as demyelination, axonal destruction or gliosis, with either steady progression and/or stepwise accumulation of focal CNS lesions. Tissue changes such as reduction of the retinal nerve fiber layer (RNFL) and the total macular volume (TMV), or brain- and spinal cord atrophy indicates an irreversible stage of tissue destruction. Whether these changes are found in all MS patients, and if there is a correlation with clinical disease state, remains controversial. The objective of our study was to determine, whether there was any correlation between the RNFL or TMV of patients with MS, and: (1) the lesion load along the visual pathways, (2) the ratios and absolute concentrations of metabolites in the normal-appearing white matter (NAWM), (3) standard brain atrophy indices, (4) disease activity or (5) disease duration.

**Methods:**

28 MS patients (RRMS, n = 23; secondary progressive MS (SPMS), n = 5) with moderately-high disease activity or long disease course were included in the study. We utilised: (1) magnetic resonance imaging (MRI) and (2) -spectroscopy (MRS), both operating at 3 Tesla, and (3) high-resolution spectral domain-OCT with locked reference images and eye tracking mode) to undertake the study.

**Results:**

There was no consistency in the pattern of CNS metabolites, brain atrophy indices and the RNFL/TMV between individuals, which ranged from normal to markedly-reduced levels. Furthermore, there was no strict correlation between CNS metabolites, lesions along the visual pathways, atrophy indices, RNFL, TMV, disease duration or disability.

**Conclusions:**

Based on the findings of this study, we recommend that the concept of ‘clinico-radiologico paradox’ in multiple sclerosis be extended to CROP–‘clinico-radiologico-ophthalmological paradox’. Furthermore, OCT data of MS patients should be interpreted with caution.

## Introduction

A current and frequent topic of ophthalmological debate is whether the reduction of the retinal nerve fiber layer (RNFL) or the total macular volume (TMV) has any consistent correlation with brain- or spinal cord atrophy, or even the brain weight of multiple sclerosis (MS) patients. If a definitive correlation does exist in the majority of MS patients, the underlying pathogenic mechanisms remain unclear, and would require elucidation. It is difficult to imagine how the 1–3 million axons of the optic nerve (which is itself part of the central nervous system (CNS)) should reflect billions of axons within the CNS. Such neuronal convergence would suggest pathological processes other than solely ‘trans-synaptical neurodegeneration’.

A correlation between brain atrophy and any other clinical or physiological parameter in MS patients has yet to be identified (e.g. [1–3]). Despite ongoing investigations, the cause of MS is unknown and each patient follows his/her individual course. Axonal injury or axonal dysfunction is generally accepted to be the pathological correlate to temporary dysfunction or disability in MS patients. But neither high field magnetic resonance imaging (MRI), magnetic resonance spectroscopy (MRS) or high-resolution spectral domain-optical coherence tomography (SD-OCT) (which allows visualisation of axonal changes in the CNS or the retina *in vivo*), enable a reliable prediction that could be applied to individual MS patients. On the contrary, more than two decades of MS research has led to the concept of ‘the clinico-radiological paradox’ and ‘radiological isolated syndrome’, as neither lesion load, brain volume/atrophy nor any other radiological parameter was found to correlate strictly to the patients’ clinical parameters [1–3].

The use of optical coherence tomography (OCT) in MS research was introduced only a few years ago, and it has evolved rapidly, making data from different OCT devices difficult to compare [4–5]. Using the following search terms: {(“optical coherence tomography” AND “brain atrophy”) OR (“optical coherence tomography” AND “brain weight”)} only 20 papers could be found using PUBMED (http://www.ncbi.nlm.nih.gov/pubmed) in March 2014 [6–25]]: 14 original research articles [6–9; 11–12; 14–16; 20–22; 24–25] and 6 reviews [10; 13; 17–19; 23]). Of note is that 272 papers could be found with other search terms {“optical coherence tomography” AND “multiple sclerosis"} in the same period from January 2006 to March 2014.

Of the 14 original research articles, only 8 were OCT studies of MS patients [6–8; 16; 20–22; 25]. The others were OCT studies of patients with spinocerebellar ataxia type 1 [12], CADASIL [15], isolated unilateral optic neuritis [14], clinical isolated syndrome [9], neuromyelitis optica [24], and one histopathological study that found pathological retinal changes which correlated to brain weight [11]. The correlations between retinal changes and several MRI-based measures of atrophy were highly variable [7–8; 16; 20–22; 25], and might be explained by the use of conventional time domain-OCT (TD-OCT) devices instead of new-generation, high-resolution SD-OCT devices [4–5] This is in addition to variability between raters and sessions [5; 26–30], measurement artefacts [29–31], inter-individual variability due to sex and age [29], and intra-individual, physiological variations of the retina [32].

A key study combining (1) stringent clinical inclusion criteria, (2) advanced high-resolution OCT, and (3) MRI/MRS technique, in order to investigate whether a ‘clinico-radiological-ophthalmological paradox’ (CROP) exists in MS has yet to be performed, and was the aim of our study.

## Material and Methods

### Participants

This study was approved by the local Ethics Committee (Commission of Medical Ethics of Vienna- Ethics Approval/Registration Numbers: EK 06-169-VK and EK-08-028-0308; and Ethical commission of the Medical University of Vienna- Ethics Approval/Registration Number: 414/2008). Informed, written consent was obtained from all patients and volunteers before entry into the study.

All MS patients included in this study (and in two previously published studies [33–34]) had to fulfil Poser’s diagnostic criteria [35], Barkhof’s MRI criteria [36] and had to have detectable oligoclonal bands [37]. 59 MS patients consented to the use of high-resolution spectral domain-OCT (SD-OCT) [33], 37 MS patients consented to MRI and magnetic resonance spectroscopy (MRS), both operating at 3 Tesla [34], but only 28 MS patients consented to both OCT and MRI/MRS. 23 patients followed a relapsing-remitting course with well-defined relapses and lack of clinical progression between relapses (RRMS, mean age, 38.9 years ± 2.4 [range, 19.75 to 61.0]; f:m = 16:7; pat. No 1–23; Table 1). Five patients followed a secondary progressive disease course (SPMS, mean age, 39.1 years ± 6.4 [range, 27.0 to 47.5]; f:m = 2:3; pat. No 24–28; Table 1).

**Table 1:** Clinical data.

				before OCT examination					in further follow-up
	MS		age at	therapy	relapses*	ON		age at	therapy was changed to
No	subtype	sex	onset			right	left	OCT/ MRI-MRS	
**1**	RRMS	f	34.5	MITOX, GLAT, IFN(b), IFN(a)	7	0	0	40.5	natalizumab
**2**	RRMS	f	18.5	IFN(a), IFN(b)	4	0	0	23.5	natalizumab
**3**	RRMS	f	36.0	MITOX^1^, IFN(a)	7	0	0	42.0	natalizumab
**4**	RRMS	f	31.5	none	3	0	0	38.0	none
**5**	RRMS	m	40.0	IFN(a)	3	0	0	45.5	none
**6**	RRMS	f	28.5	IFN(b)	3	0	0	39.0	natalizumab
**7**	RRMS	f	43.0	GLAT, IFN(b), none^2^	4	0	0	48.0	natalizumab
**8**	RRMS	f	40.0	none	2	0	0	42.25	none
**9**	RRMS	m	24.0	none	2	0	0	25.0	none
**10**	RRMS	f	18.0	GLAT, none^3^	2	0	0	19.75	none
**11**	RRMS	f	29.75	IFN(a)^4^, none	4	0	0	36.0	none
**12**	RRMS	m	31.0	IFN(b)	2	0	0	33.25	IFN(b)
**13**	RRMS	m	51.0	IFN(b)	2	0	0	52.0	IFN(b)
**14**	RRMS	m	27.5	GLAT	4	0	0	39.0	GLAT
**15**	RRMS	f	30.0	IFN(b)^5^, none	4	0	0	46.0	none
**16**	RRMS	m	39.0	IFN(c)	4	0	0	45.0	IFN(c)
**17**	RRMS	f	16.0	GLAT	4	0	0	61.0	GLAT
**18**	RRMS	f	26.0	IFN(a), IFN(b), MITOX^6^, none	9	1	1	32.0	none
**19**	RRMS	f	17.75	IFN(a), IFN(b)	6	1	3	19.75	natalizumab
**20**	RRMS	f	31.0	IFN(a), IFN(b)	4	1	0	36.0	IFN(b)
**21**	RRMS	f	20.0	IFN(b)	8	1	1	47.5	IFN(b)
**22**	RRMS	m	22.5	GLAT, IFN(a), IFN(b), natalizumab	10	0	1	42.5	natalizumab
**23**	RRMS	f	20.0	IFN(a)	3	0	4	41.0	IFN(a)
**24**	SPMS	m	40.0	GLAT, MITOX^7^, none	3	0	0	46.5	none
**25**	SPMS	f	13.0	IFN(b), MITOX^8^, none	5	0	0	27.0	none
**26**	SPMS	m	25.0	IFN(c), GLAT, IFN(a), IFN(b)	10	1	1	47.5	IFN(b)
**27**	SPMS	m	22.0	IFN(b)	5	1	0	30.5	none
**28**	SPMS	f	16.0	IFN(a), MITOX^9^, none	6	0	2	44.25	none

**ON**, **optic neuritis;**

*, relapses treated with high dose steroid pulse therapy; no included patient had an ON within 12 months prior to the beginning of the study;

**GLAT**, glatiramer-acetate 20mg subcutaneous once daily; **MITOX**, mitoxantrone; **IFN(a)**, interferon beta 1a intramuscularly once per week; **IFN(b)**, interferon beta 1a (44μg) subcutaneous trice per week; **IFN(c)**, interferon beta 1b (250μg) subcutaneous alternate day. Most importantly, the disease activity remained high in further follow-up with a median observation period of 22 ± 0.5 months [33]. However, no significant reduction of either the RNFL or the TMV could be found in follow-up [33; 36].

^1^, discontinued (48mg mitoxantrone per m^2^ body surface); **none**, neither specific immunomodulatory or immunsuppressive therapy, drug holiday;

^2^, drug withdrawal 12 months before OCT examination;

^3^, drug withdrawal 6 months before OCT examination;

^4^, drug withdrawal 20 months before OCT examination;

^5^, high titres of anti-interferon autoantibodies, drug withdrawal 14 months before OCT examination;

^6^, mitoxantrone cumulative dose 96mg per m^2^ body surface, drug withdrawal 10 months before OCT examination;

^7^, mitoxantrone cumulative dose 92mg per m^2^ body surface, drug withdrawal 10 months before OCT examination;

^8^, mitoxantrone cumulative dose 92mg per m^2^ body surface, drug withdrawal 26 months before OCT examination;

^9^, mitoxantrone cumulative dose 108mg per m^2^ body surface, drug withdrawal 27 months before 1^st^ OCT examination.

All MS patients had their MRI/MRS and OCT examination on the same day, one scan immediately after the other. Prior to investigations, hydration of patients (a confounding factor) was ensured by the intake of sufficient amounts of water (approximately 1 to 2 litres). Detailed patient demographic and clinical data has been described previously [38–39] and again in Table 1. Patients were treated with beta-interferons, glatiramer-acetate, mitoxantrone and natalizumab (Table 1).

### MRI and MRS

The standardized MRI/MRS protocols were carried out as described previously [34].

### Analysis of Lesion load along the visual pathways

Lesion regions were analyzed with T_2_ -TIRM axial multi slice MR images with a spatial resolution of 0.45 x 0.45 x 4 mm, using the medical imaging software package Jim (Xinapse Systems Ltd, Aldwincle, UK), by two independent assessors (IK, LV) with experience in MRI image analysis. Image slices were inspected between the first appearance of the inferior horns of lateral ventricles, and the point where the posterior horns of lateral ventricles lost their sharpness. Lesions were semi-automatically selected along the visual pathways. Assessors manually located lesions based on the difference in signal intensity between lesions and surrounding tissue, with the software automatically outlining the entire lesion. In the case where one lesion was also covering the area outside the visual pathway, those parts of the lesion were manually excluded. For regional quantification purposes the lesions were divided into four groups according to their location in the brain (anterior dextra, anterior sinistra, posterior dextra and posterior sinistra). The posterior edge of the inferior horn of lateral ventricle was set as the border between posterior and anterior regions. The volume of the brain in the same slice range was also selected semi-automatically. The cavities and ventricles were excluded from the whole brain volume. The total lesion load (defined as the lesion volume (ml) over brain volume (ml)) in all evaluated slices, and regional lesion load (lesion load per quadrant) were subsequently calculated. For each parameter, the mean was calculated from two independent examinations performed by two assessors (IJK, VK) who were blind to the data of each other, and without knowledge of the clinical and OCT data.

### Analysis of Brain Atrophy

An experienced examiner (HR), without knowledge of the clinical and OCT data, determined the following established indices to quantify global and focal, cortical and subcortical atrophy [40–42]: 1) the Evans ratio; 2) the caudate head index (CHI); 3) the basal cistern index (BCI); 4) the cella media index (CMI); 5) the maximum width of the third ventricle; 6) the maximum width of the fourth ventricle; 7) the maximum width of the anterior interhemispheric fissure (MIF); 8) the maximum width of the Sylvian fissure (MSF); and 9) the maximum frontal subarachnoid space (MFSS).

### High Resolution spectral domain OCT

We utilised a high resolution SD-OCT which combines OCT technology with a confocal scanning laser ophthalmoscope (Heidelberg Engineering, Heidelberg, Germany, Spectralis software version 4.0.3.0, Eye Explorer Software 1.6.1.0). A special eye-tracking mode (TrueTrack^™^) and high-scanning speed allows the reduction of artefacts due to eye movement. Each peripapillary OCT is registered and locked to a reference image. OCT software can identify previous scan locations and “guide” the OCT laser beam to scan the identical location again. To optimize the signal-to-noise ratio and image quality, 16 frames (B scans) of the same scanning position were averaged with the Automatic Real-Time averaging mode (ART mode). All RNFL scans were acquired in the high-resolution acquisition mode allowing a more detailed differentiation of retinal layers, with pupil dilation.

Furthermore, signal strength has been shown to affect RNFL thickness measurements using conventional Stratus OCT [33; 38]. Therefore, scans with low quality (signal strength for RNFL and TMV scans < 15) and failing RNFL segmentation were excluded. Measurements were repeated until a technically-excellent quality was achieved. Criteria for determining scan quality included: (1) a clear fundus image before and during image acquisition, (2) absence of scan or algorithm failures, (3) even and dense grey scale saturation throughout all retinal layers with dense grey visible in the RPE, and (4) the RNFL visible without missing or blank areas and a continuous scan pattern.

All automated measurements of macular thickness and volume were performed through dilated pupils with a high-resolution macular scan protocol allowing for a more detailed differentiation of retinal layers (the TMV compounds of inner limiting membrane, nerve fiber layer, ganglion cell layer, inner plexiform layer, inner nuclear layer, outer plexiform layer, outer nuclear layer, external limiting membrane, photoreceptor layer, and retinal pigment epithelium).

The SD-OCT imaging protocol comprised 49 B-scans per volume scan of 20° × 20°, and each scan was averaged with 9 frames per B-scan. Topographic macular surface maps were constructed automatically by the OCT software and displayed with numeric averages of the mean thickness for each of the nine map sectors (F, foveal; TI, inner temporal; TO, outer temporal; II, inner inferior; IO, outer inferior; NI, inner nasal; NO, outer nasal; SI, inner superior; SO, outer superior) within three concentric regions of 1, 3, and 6 mm diameter, respectively, as defined by the Early Treatment Diabetic Retinopathy Study (ETDRS).

All RNFL and macular scans were performed several times by one skilled and trained observer (NS) within one session until at least 3 high-quality scans were achieved and used for further analysis. The observer had no knowledge of clinical data or specific baseline and MRI/MRS data. Final analysis was only performed on scans without segmentation errors, and no manual correction was performed in any case. Normal ranges of RNFL thickness and TMV was determined by the internal database of the OCT device and compared to published reports and prior experience [4; 26].

Patients with other ocular pathologies that may reduce RNFL thickness such as glaucoma, anterior ischemic optic neuropathy, high myopia, and congenital abnormalities of the optic nerves etc., were excluded.

### Statistics

This was a prospective study. We used descriptive and analytic statistics as previously described in detail [33; 38]. Briefly, nonparametric tests (Mann-Whitney, Kolmogorov-Smirnov, Bonferroni-Holm, Chi-squared) and regression analysis were applied (statgraphics plus 5.1). All test results were considered significant if p-values were below 0.05. All parameters are expressed as means, medians, minimums and maximums and standard error of the mean. As case numbers are rather low, the data has been presented in detailed surveys (Tables 1–5) and in an individual manner to overcome the limitations of group comparisons (see cases 1–5 in the results and references [33; 38–39]).

**Table 2:** Metabolites in NAWM and lesion load in the visual pathways in MS patients.

					Metabolites in NAWM			lesion volume in visual pathway				
Group	N =	f:m	age	disease	Cho	Cr[mM]	NAA[mM]	AD/	AS/	PD/	PS/	Total
				duration	[mM]	[mM]	[mM]	Volume	Volume	Volume	Volume	Lesion/
												Volume
**RRMS**	**17**	**11:6**	**39.75**	**8.1**	**2.5**	**8.3**	**11.3**	**0.04**	**0.05**	**0.48**	**0.46**	**1.03**
**without ON**												
STE			± 2.5	± 2.5	± 0.10	± 0.30	± 0.60	± 0.02	± 0.02	± 0.09	± 0.08	± 0.2
range			19.75–61.0	1.0–45.0	1.47–3.15	4.72–10.55	4.86–14.08	0.00–0.28	0.00–0.23	0.01–1.39	0.02–1.22	0.03–2.71
**RRMS**	**6**	**5:1**	**36.5**	**13.6**	**2.4**	**8.4**	**11.9**	**0.10**	**0.13**	**0.90**	**0.78**	**1.91**
**with ON**												
STE			± 4.0	± 4.3	± 0.07	± 0.44	± 1.09	± 0.06	± 0.05	± 0.20	± 0.23	± 0.51
range			19.75–47.5	2.0–27.5	2.11–2.58	6.48–9.48	7.67–14.95	0.00–0.33	0.00–0.28	0.19–1.48	0.10–1.47	0.32–3.54
**SPMS**	**2**	**1:1**	**36.75**	**10.25**	**2.2**	**7.4**	**9.9**	**0.30**	**0.33**	**1.58**	**0.96**	**3.17**
**without ON**												
STE			± 9.75	± 3.75	± 0.28	± 0.45	± 0.73	± 0.10	± 0.10	± 0.54	± 0.06	± 0.80
range			27.0; 46.5	6.5; 14.0	1.91–2.48	6.91–7.81	9.14–10.59	0.20–0.40	0.23–0.42	1.04–2.12	0.90–1.02	2.37; 3.54
**SPMS with ON**	**3**	**1:2**	**40.75**	**19.75**	**2.55**	**8.4**	**9.6**	**0.31**	**0.15**	**1.24**	**0.95**	**2.65**
**with ON**												
STE			± 5.2	± 5.9	± 0.11	± 0.21	± 0.81	± 0.13	± 0.06	± 0.23	± 0.34	± 0.69
range			30.5–47.5	8.5–28.3	2.40–2.76	8.11–8.77	8.25–11.07	0.05–0.49	0.04–0.24	0.78–1.48	0.48–1.60	1.36–3.70

**Metabolites**, N-Acetyl-Aspartate (NAA), Choline (Cho) and creatine (Cr) given in mM; **lesion load in the visual pathways**, here given as ratio of lesion volume in the visual pathways (AD, right anterior; AS, left anterior; PD, right posterior; PS, left posterior and total lesion volume) to total brain volume.

**Table 3:** Brain atrophy indices in MS patients.

Group	N =	f:m	age	disease	Evans	CHI	CMI	BCI	maximum	maximum	MFSS	MIF	MSF
									width	width			
				duration	ratio				of 3^rd^	of 4^th^			
									ventricle	ventricle			
**RRMS**	**17**	**11:6**	**39.75**	**8.1**	**0.26**	**0.12**	**0.19**	**0.22**	**5.5**	**11.2**	**2.5**	**2.4**	**2.0**
**without ON**													
STE			± 2.5	± 2.5	± 0.1	± 0.1	± 0.2	± 0.005	± 0.73	± 0.37	± 0.2	± 0.2	± 0.2
range			19.75–61.0	1.0–45.0	0.17–0.34	0.05–0.21	0.1–0.33	0.17–0.25	1.5–11.1	9.3–14.0	1.0–4.1	1.5–4.3	1.0–5.0
**RRMS**	**6**	**5:1**	**36.5**	**13.6**	**0.23**	**0.08**	**0.12**	**0.22**	**3.6**	**10.8**	**3.1**	**2.5**	**2.2**
**with ON**													
STE			± 4.0	± 4.3	± 0.1	± 0.1	± 0.2	± 0.01	± 0.59	± 0.96	± 0.4	± 0.3	± 0.5
range			19.75–47.5	2.0–27.5	0.21–0.25	0.05–0.12	0.07–0.21	0.19–0.24	1.7–5.2	7.3–13.5	2.0–4.0	1.3–3.4	1.0–4.0
**SPMS**	**2**	**1:1**	**36.75**	**10.25**	**0.26**	**0.12**	**0.25**	**0.26**	**7.3**	**10.3**	**3.40**	**3.8**	**2.8**
**without ON**													
STE			± 9.75	± 3.75	± 0.1	± 0.2	± 0.2	± 0.04	± 1.4	± 0.3	± 0.7	± 0.5	± 0.8
range			27.0; 46.5	6.5; 14.0	0.25–0.27	0.10–0.13	0.23–0.27	0.22–0.31	5.9–8.7	10.0–10.6	2.7–4.1	3.3–4.3	2.0–3.6
**SPMS**	**3**	**1:2**	**40.75**	**19.75**	**0.23**	**0.10**	**0.16**	**0.19**	**5.5**	**11.4**	**2.8**	**1.9**	**2.3**
**with ON**													
STE			± 5.2	± 5.9	± 0.2	± 0.1	± 0.2	± 0.02	± 1.72	± 1.0	± 0.1	± 0.4	± 0.5
range			30.5–47.5	8.5–28.3	0.20–0.25	0.08–0.12	0.12–0.19	0.16–0.22	3.6–8.9	9.4–12.7	2.7–3.0	1.2–2.5	1.4–2.8

**CHI**, the caudate head index; **BCI**, the basal cistern index; **CMI**, the cella media index; **MIF**, the maximum width of the anterior interhemispheric fissure; **MSF**, the maximum width of the Sylvian fissure; and **MFSS**, the maximum frontal subarachnoid space.

**Table 4:** RNFL and TMV in MS patients, global.

Group	N =	f:m	age	disease	RNFL,	RNFL,	TMV,	TMV,
				duration	global OD	global OS	OD	OS
**RRMS**	**17**	**11:6**	**39.75**	**8.1**	**100.24**	**97.29**	**8.43**	**8.33**
**without ON**								
STE			± 2.5	± 2.5	± 2.70	± 0.2.68	± 0.12	± 0.14
range			19.75–61.0	1.0–45.0	73.0–124.0	73.0–120.0	7.71–9.39	7.32–9.43
**RRMS**	**6**	**5:1**	**36.5**	**13.6**	**84.67**	**87.83**	**8.18**	**8.34**
**with ON**								
STE			± 4.0	± 4.3	± 3.27	± 3.48	± 0.23	± 0.20
range			19.75–47.5	2.0–27.5	74.0–97.0	77.0–100.0	7.47–9.02	7.82–9.15
**SPMS**	**2**	**1:1**	**36,75**	**10.25**	**83.00**	**82.00**	**8.15**	**8.51**
**without ON**								
STE			± 9.75	± 3.75	± 2.00	± 4.00	± 0.11	± 0.32
range			27.0; 46.5	6.5; 14.0	81.0–85.0	78.0–86.0	8.04–8.26	8.19–8.83
**SPMS**	**3**	**1:2**	**40.75**	**19.75**	**99.33**	**83.67**	**8.10**	**7.95**
**with ON**								
STE			± 5.2	± 5.9	± 9.28	± 10.71	± 0.05	± 0.36
range			30.5–47.5	8.5–28.3	81.0–111.0	66.0–103.0	8.01–8.18	7.33–8.57

**OD**, right eye; **OS**, left eye. **All RNFL values are given in μm. All TMV values are given in mm** ^**3**^.

**Table 5:** RNFL in MS patients.

Group	G	G	T	TS	TI	S	I	N	NS	NI	T	TS	TI	S	I	N	NS	NI
	OD	OS	OD	OD	OD	OD	OD	OS	OS	OS	OS	OS	OS	OS	OS	OD	OD	OD
**RRMS**	**100.2**	**97.3**	**66.1**	**128.3**	**143.1**	**122.2**	**131.7**	**67.5**	**113.9**	**111.0**	**71.7**	**132.4**	**140.2**	**123.7**	**125.6**	**79.8**	**115.9**	**122.5**
**without ON**																		
STE	± 2.7	± 2.6	± 3.0	± 4.2	± 6.3	± 5.6	± 4.9	± 2.9	± 6.3	± 6.7	± 2.9	± 4.9	± 5.3	± 4.2	± 4.9	± 4.8	± 8.9	± 7.2
range	73–124	73–120	40–90	90–162	91–191	74–192	94–168	47–96	76–193	75–163	45–88	85–159	105–172	99–170	93–162	51–131	58–221	79–168
**RRMS**	**84.7**	**87.8**	**51.3**	**121.8**	**118.2**	**110.8**	**107.2**	**66.3**	**112.5**	**98.3**	**58.0**	**120.8**	**126.0**	**116.1**	**111.4**	**69.0**	**103.5**	**99.5**
**with ON**																		
STE	± 3.3	± 3.5	± 3.6	± 7.8	± 4.6	± 8.1	± 4.4	± 6.3	± 10.5	± 8.1	± 6.3	± 10.4	± 11.9	± 8.3	± 6.3	± 6.6	± 12.8	± 6.8
range	74–97	77–100	40–67	105–148	110–139	80–133	96–125	50–87	77–150	64–118	41–86	92–165	75–159	85–143	90–125	50–88	55–151	74–118
**SPMS**	**83.0**	**82.0**	**51.5**	**118.5**	**114.5**	**112.5**	**93.5**	**62.0**	**85.0**	**82.5**	**61.0**	**112.0**	**126.5**	**98.5**	**104.5**	**75.0**	**105.5**	**72.5**
**without ON**																		
STE	± 2.0	± 4.0	± 1.5	± 8.5	± 1.5	± 1.5	± 5.5	± 3.0	± 16.0	± 4.5	± 6.0	± 19.0	± 0.5	± 17.5	± 1.5	± 0.0	± 5.5	± 12.5
range	81–85	78–86	50–53	110–127	113–116	111–114	88–99	59–65	69–101	78–87	55–67	93–131	126–127	81–116	103–106	75–75	100–111	60–85
**SPMS**	**99.3**	**83.7**	**69.3**	**143.7**	**145.0**	**131.7**	**121.2**	**55.0**	**99.3**	**100.0**	**56.7**	**126.7**	**117.0**	**113.2**	**108.5**	**71.3**	**119.3**	**97.0**
**with ON**																		
STE	± 9.3	± 10.7	± 7.8	± 15.9	± 18.7	± 9.4	± 8.8	± 6.4	± 4.8	± 18.2	± 14.3	± 5.9	± 17.9	± 4.8	± 17.2	± 10.4	± 8.3	± 10.2
range	81–111	66–103	54–79	112–162	118–181	114–146	104–133	43–65	94–109	66–128	36–84	115–134	93–152	105–122	80–140	51–85	107–135	84–117

**Retinal sectors: OD**, right eye; **OS**, left eye; **G**, global; **S**, superior; **I**, inferior; **T**, temporal; **TS**, temporal superior; **TI**, temporal inferior; **N**, nasal; **NS**, nasal superior; **NI**, nasal inferior. All values are given in μm.

## Results

Our results may be summarized briefly as follows: We found inter-individual differences in clinical characteristics, NAA levels of the NAWM, the lesion load in the visual pathways, brain atrophy indices and both RNFL thickness and the TMV (data not shown). The levels ranged from normally to markedly reduced levels.

**Fig 1:**
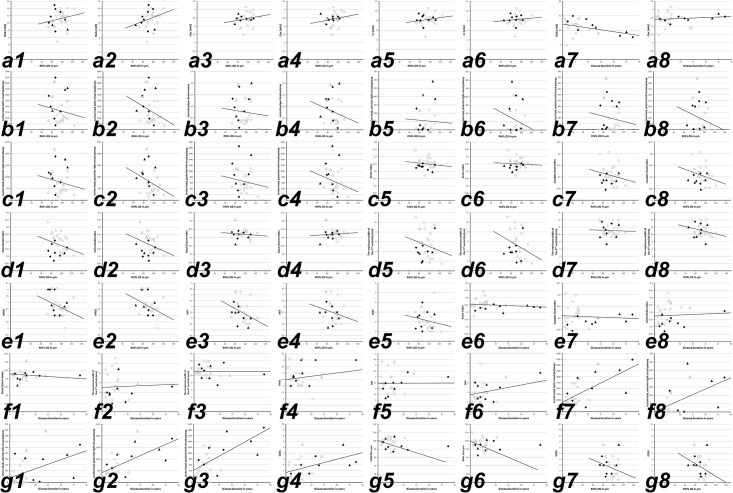
**white squares**, RRMS without ON; **black squares**, RRMS with ON; **white triangles**, SPMS without ON; **black triangles**, SPMS with ON; **black line**, linear regression curve. *Abbreviations*: **OD**, oculus dexter (right eye); **OS**, oculus sinister (left eye); **RNFL**, retinal nerve fiber layer; **NAA**, N-acetyl-aspartate; **Cho**, choline; **Cr**, creatine; **NAWM**, normal appearing white matter; **MIF**, the maximum width of the anterior interhemispheric fissure; **MSF**, the maximum width of the Sylvian fissure; **MFSS**, the maximum frontal subarachnoid space; **EDSS**, expanded disability severity scale. **a1**-**g8**, linear regression curves for: **a1**, RNFL vs. NAA (right eye); **a2**, RNFL vs. NAA (left eye); **a3**, RNFL vs. Cho (right eye); **a4**, RNFL vs. Cho (left eye); **a5**, RNFL vs. Cr (right eye); **a6**, RNFL vs. Cr (left eye); **a7**, disease duration vs. NAA in the NAWM; **a8**, disease duration vs. Cho; **b1**, RNFL vs. lesion load (right eye); **b2**, RNFL vs. lesion load (left eye); **b3**, RNFL vs. lesion load per brain volume (right eye); **b4**, RNFL vs. lesion load per brain volume (left eye); **b5**, RNFL vs. lesion load along anterior right visual pathway (right eye); **b6**, RNFL vs. lesion load anterior right visual pathway (left eye); **b7**, RNFL vs. lesion load along anterior left visual pathway (right eye); **b8**, RNFL vs. lesion load anterior left visual pathway (left eye); **c1**, RNFL vs. lesion load along posterior right visual (right eye); **c2**, RNFL vs. lesion load along posterior left visual pathway (left eye); **c3**, RNFL vs. lesion load along posterior left visual (right eye); **c4**, RNFL vs. lesion load along posterior left visual pathway (left eye); **c5**, RNFL vs. Evan’s Index (right eye); **c6**, RNFL vs. Evan’s Index (left eye); **c7**, RNFL vs. Caudate Head Index (right eye); **c8**, RNFL vs. Caudate Head Index Index (left eye); **d1**, RNFL vs. Cella Media Index (right eye); **d2**, RNFL vs. Cella Media Index (left eye); **d3**, RNFL vs. Basal Cistern Index (right eye); **d4**, RNFL vs. Basal Cistern Index Index (left eye); **d5**, RNFL vs. the maximum width of the 3^rd^ ventricle (right eye); **d6**, RNFL vs. the maximum width of the 3^rd^ ventricle (left eye); **d7**, RNFL vs. the maximum of the 4^th^ width ventricle (right eye); **d8**, RNFL vs. the maximum of the 4^th^ width ventricle (left eye); **e1**, RNFL vs. MFSS (right eye); **e2**, RNFL vs. MFSS (left eye); **e3**, RNFL vs. MIF (right eye); **e4**, RNFL vs. MIF (left eye); **e5**, RNFL vs. MSF (right eye); **e6**, RNFL vs. MSF (left eye); **e7**, disease duration vs. Evan’s Index; **e8**, disease duration vs. Caudate Head Index; **f1**, disease duration vs. Cella Media Index; **f2**, disease duration vs. the maximum width of the 3^rd^ ventricle; **f3**, disease duration vs. the maximum width of the 4^th^ ventricle; **f4**, disease duration vs. MFSS; **f5**, disease duration vs. MIF; **f6**, disease duration vs. MSF; **f7**, disease duration vs. lesion load along both visual pathways; **f8**, disease duration vs. lesion load along the anterior right visual pathway; **g1**, disease duration vs. lesion load along the anterior left visual pathway; **g2**, disease duration vs. lesion load along the posterior right visual pathway; **g3**, disease duration vs. lesion load along the posterior left visual pathway; **g4**, disease duration vs. EDSS; **g5**, disease duration vs. RNFL (right eye); **g6**, disease duration vs. RNFL (left eye); **g7**, RNFL (right eye) vs. EDSS; **g8**, RNFL (right eye) vs. EDSS. Regression analyses demonstrated only weak correlations between the examined parameters a1-g8 of all 28 MS patients included in this study and associated subgroups (RRMS without ON, RRMS with ON, SPMS without ON, SPMS with ON). Of note, the plotted linear regression curves in a1 –g8 are calculated for the analysis of all included MS patient.

Neither the RNFL nor TMV was found to correlate strictly with: (1) brain metabolites in the NAWM (NAA, Cho and Cr) or (2) the various brain atrophy indices and lesion load in the visual pathways. Evan’s ratio, CHI, CMI, BCI were found to be within normal range in all 28 patients even though global brain atrophy was detectable on MRI. This may suggest that these indices were not applicable to the brain atrophy seen in the MS patients in this study (Table 3). The atrophy detected in MS patients seemed to be described better by other parameters such as the maximum width of 3^rd^ and 4^th^ ventricles, MIF, MSF and MFSS (see results and Table 1, patient 1, 24 and 25, Fig 1). Usually the whole brain volume and white and grey matter volume/fractions are determined in MRT studies of MS patients to detect even mild brain atrophy. Also in most of our included MS patients there could at least a mild brain atrophy be detected by inspection of his/her MRI by a skilled radiologist and or neurologist. Interestingly, also in those MS patients with marked brain atrophy also the RNFL or TMV were not significantly reduced (this applies also for the further 2 years follow-up [38; 39]). However, we state clearly that the brain atrophy indices—we have used here—have potential methodological limitations and basically do not correlate strictly with disease course or clinical progression in all MS patients as this has been shown for so many other MRI parameters before [1].

Most importantly, there was no correlation between individual specific sectors of the retina (6 retinal sectors and globally (Table 5)) and the lesion load of their corresponding visual pathways. Correlations and possible associations were analysed for total groups of RRMS and SPMS patients (Table 6), but checked intra-individually for all 28 MS patients for plausibility and stringency. This means that the values for each patient were intentionally broken down and traced back, i.e. each individual RNFL value (global or the corresponding sector) has been analysed set to the corresponding visual pathway lesion load, the brain metabolite levels and brain atrophy indices. Nevertheless, we generally did not find consistent patterns that could suggest retro- or anterograde trans-synaptical degeneration, either in patients with, or more importantly, in patients without previous ON. (It should however be noted that our study design did not allow the measurement of whole brain volume and the parenchymal fractions for the white and grey matter, and tractography of the posterior visual pathway.)

**Table 6:** Regression Analysis. Simple regression–linear model: Independent variable, RNFL; dependent variables, NAA, N-acetyl-aspartate; Cho, choline; Cr, creatine; LL per BV, lesionload per Brain Volume, LL AR, lesion load along anterior right visual pathway; LL AL, lesion load along anterior left visual pathway; LL PR, load along posterior right visual pathway; LL PL, load along posterior left visual pathway; Evan’s Index; CHI; CMI; BCI; the maximum width of the 3^rd^ ventricle; the maximum width of the 4^th^ ventricle; MIF, the maximum width of the anterior interhemispheric fissure; MFSS, the maximum frontal subarachnoid space; MSF, the maximum width of the Sylvian fissure; DD, disease duration; EDSS, expanded disability severity scale. **1** ^**st**^ **row**: all right eyes (n = 28; with and without ON) of all included MS patient. **2** ^**nd**^ **row**: all left eyes (n = 28; with and without ON) of all included MS patient. **3** ^**rd**^ **row**: all right eyes of MS patients who never experienced an ON (neither on their right nor on their left eye; RRMS, n = 17, SPMS, n = 2; Table 1). **4** ^**th**^ **row**: all left eyes of MS patients who never experienced an ON (neither on their left nor on their right eye; RRMS, n = 17, SPMS, n = 2; Table 1). Patients are the same as in the 3^rd^ row. **5** ^**th**^ **row**: right eyes of 6 MS patients who experienced an ON on their right eyes (note, 4 out of 6 experienced ON on both eyes, 2 only on their right eyes; Table 1). **6** ^**th**^ **row**: left eyes of 6 MS patients who experienced an ON on their left eyes (note, 4 out of 7 experienced ON on both eyes, 3 only on their left eyes Table 1). For each analysis the correlation coefficient (corr. coeff.), R-squared (percent), the standard error of estimate (STE of Est.) and the p-value (analysis of variance, ANOVA) is given. Since the p-value in the ANOVA table is less than 0.01, there is a statistically significant relationship between the maximum width of the 4^th^ ventricle and the RNFL (for all patients’ right eyes, n = 28, 1^st^ row and for all patient’s left eyes, who never experienced ON, n = 17, 4^th^ row) at 99% confidence level. However, the low correlation coefficient indicates that there is only a weak relationship between the variables. R-squared statistic indicates that the simple/linear regression explains only 24.92% (1^st^ row) or 28.68% (4^th^ row) of the variability of the independent variable. In all other analyses presented here (and performed for the six OCT-Sectors, see material and methods or Table 5) no statistically significant correlation could be found (data not shown).

RNFL		NAA	Cho	Cr	LL, per	LL, AR	LL, AL	LL, PR	LL, PL	Evans	CHI	CMI	BCI	width	width	MFSS	MIF	DD	EDSS
					BV					ratio				of 3^rd^	of 4^th^				
														ventricle	ventricle				
**right eye**	**Corr. Coeff.**	**0.041**	**0.059**	**0.136**	**0.151**	**0.010**	**0.241**	**0.029**	**0.084**	**0.067**	**0.123**	**0.236**	**-0.133**	**0.347**	**0.492**	**-0.187**	**0.061**	**-0.204**	**-0.279**
**n = 28**	R-squared	0.17	0.35	1.87	2.30	0.01	5.79	0.088	0.712	0.450	1.527	5.616	1.785	12.1	24.29	3.511	0.371	4.17	7.28
	STE of Est.	2.41	0.36	1.12	0.58	168	123	566.2	451	0.05	0.04	0.07	0.02	2.63	1.463	0.79	0.87	7.11	1.71
	**p-Value**	**0.83**	**0.76**	**0.49**	**0.44**	**0.96**	**0.21**	**0.88**	**0.67**	**0.73**	**0.53**	**0.22**	**0.49**	**0.07**	**0.007**	**0.34**	**0.75**	**0.30**	**0.16**
**left eye**	**Corr. Coeff.**	**0.115**	**0.076**	**0.150**	**0.240**	**0.201**	**0.313**	**0.203**	**0.257**	**-0.043**	**-0.076**	**0.090**	**-0.044**	**0.150**	**0.462**	**-0.092**	**-0.092**	**-0.312**	**-0.373**
**n = 28**	R-squared	1.32	0.58	2.26	5.76	4.05	9.81	4.12	6.63	0.19	0.57	0.81	0.19	2.26	21.40	0.86	0.84	9.77	13.97
	STE of Est.	2.42	0.36	1.12	0.57	164.9	120.8	554.7	437.6	0.05	0.04	0.07	0.02	2.78	1.49	0.80	0.87	6.90	1.65
	**p-Value**	**0.56**	**0.67**	**0.45**	**0.22**	**0.30**	**0.10**	**0.30**	**0.20**	**0.83**	**0.70**	**0.65**	**0.83**	**0.44**	**0.01**	**0.63**	**0.64**	**0.1**	**0.05**
**right eye,**	**Corr. Coeff.**	**0.151**	**0.05**	**0.31**	**-0.006**	**0.068**	**0.111**	**-0.043**	**-0.102**	**0.200**	**0.063**	**0.297**	**-0.188**	**0.329**	**0.387**	**-0.262**	**0.030**	**-0.248**	**-0.162**
**without ON**	R-squared	2.30	0.23	9.76	0.01	0.47	1.23	0.19	1.05	4.01	0.40	8.86	3.52	10.88	14.92	6.89	0.09	6.13	2.63
**n = 19**	STE of Est.	2.44	0.42	1.21	0.54	119.2	125,0	525,3	334.8	0.05	0.04	0.07	0.02	2.86	1,40	0.75	0.93	7.39	1.29
	**p-Value**	**0.54**	**0.84**	**0.20**	**0.97**	**0.78**	**0.61**	**0.86**	**0.68**	**0.41**	**0.80**	**0.21**	**0.44**	**0.17**	**0.10**	**0.28**	**0.90**	**0.31**	**0.51**
**left eye**	**Corr. Coeff.**	**0.257**	**0.078**	**0.335**	**0.002**	**0.060**	**0.082**	**-0.027**	**0.031**	**0.233**	**-0.073**	**0.244**	**-0.018**	**0.198**	**0.535**	**-0.250**	**-0.087**	**-0.387**	**-0.227**
**without ON**	R-squared	6.60	0.60	11.26	0.001	0.36	0.69	0.07	0.10	5.45	0.54	5.97	0.03	3.93	28.68	6.26	0.75	14.94	5.16
**n = 19**	STE of Est.	2.39	0.43	1.19	0.53	120,59	125,38	525.60	336,47	0.05	0.04	0.07	0.02	2.97	1.28	0.75	0.94	7.04	1.27
	**p-Value**	**0.29**	**0.75**	**0.16**	**0.10**	**0.81**	**0.74**	**0.91**	**0.90**	**0.34**	**0.76**	**0.31**	**0.94**	**0.42**	**0.02**	**0.30**	**0.72**	**0.10**	**0.35**
**right eye**	**Corr. Coeff.**	**0.407**	**0.126**	**0.077**	**-0.094**	**-0.387**	**0.302**	**-0.277**	**-0.293**	**0.333**	**0.221**	**-0.333**	**-0.333**	**0.107**	**0.686**	**-0.386**	**0.133**	**-0.687**	**-0.690**
**with ON**	R-squared	16.60	1.60	0.59	0.87	15.01	9.12	7.69	8.57	11.12	4.87	11.12	11.12	1.15	46.92	14.89	1.78	47.21	47.65
**n = 6**	STE of Est.	2.06	2.32	0.63	0.41	241.75	119.20	433.31	349.74	0.04	0.04	0.04	0.04	1.15	1.47	0.86	0.92	3.41	2.04
	**p-Value**	**0.42**	**0.81**	**0.88**	**0.86**	**0.45**	**0.56**	**0.59**	**0.57**	**0.51**	**0.67**	**0.52**	**0.52**	**0.84**	**0.13**	**0.45**	**0.80**	**0.13**	**0.13**
**left eye**	**Corr. Coeff.**	**-0.262**	**0.550**	**-0.515**	**0.354**	**0.134**	**0.627**	**0.361**	**0.286**	**-0.387**	**0.236**	**0.426**	**0.123**	**0.309**	**0.662**	**-0.129**	**0.041**	**-0.393**	**-0.504**
**with ON**	R-squared	6.84	30.26	26.49	12.59	1.80	39.26	13.09	8.18	14.99	5.59	18.18	1.56	9.53	43.87	1.66	0.17	15.44	25.35
**n = 7**	STE of Est.	2.95	0.15	0.82	0.72	255.51	101.97	628.24	681.43	0.039	0.04	0.04	0.04	2.49	1.67	0.92	0.72	7.36	2.25
	**p-Value**	**0.57**	**0.21**	**0.24**	**0.43**	**0.77**	**0.13**	**0.43**	**0.53**	**0.39**	**0.61**	**0.34**	**0.79**	**0.50**	**0.10**	**0.78**	**0.93**	**0.38**	**0.25**

On the other hand, each MS patient seemed to follow his own, very individual pattern. In order to make this clear, we present very briefly five patients (2 RRMS without ON, 1 RRMS with ON, 1 SPMS patient with ON and 1 SPMS without ON).

**Fig 2:**
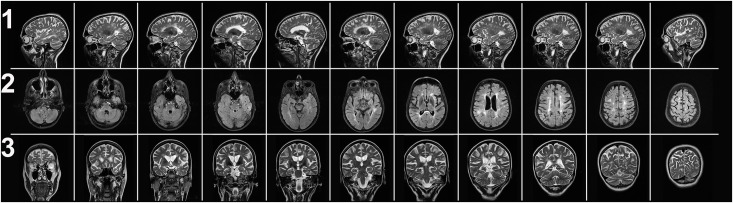
CROP, Clinico-Radiologico-Ophthalmological Paradox in MS. MRI, 3 Tesla, serial sections: 1^st^ row, sagittal, 2^nd^ row, axial and 3^rd^ row, coronar images from a RRMS patient with rather long disease course and highly active disease. Although very high lesion load in the whole brain and visual pathway and obvious brain atrophy the patient had normal RNFL and TMV values. (patient 1, Table 1).

Firstly, a female RRMS patient without previous ON and moderately long disease duration (6 years), but (1) very active disease course, (2) highest visual pathway—lesion load in the RRMS group without ON, (3) lowest ratio of NAA to Cr (1.46) and second-lowest absolute concentration of NAA in her NAWM (7.08mM), and (4) signs of brain atrophy with highest values for the maximum width of her 3^rd^ and 4^th^ ventricles (9.5 and 14.0mm), MFSS (3.0), MIF (4.0) and MSF (5.0), showed normal values for RNFL and TMV. In other words, one would expect very low RNFL and TMV values in this patient, not the opposite, i.e. normal values, which are partly the highest of all included MS patients (RNFL global, right eye, 110 μm; left eye, 101μm). The TMV-values were 8.0mm³ (right eye) and 8.36 mm³ (left eye). This patient had an EDSS of 3.0. Before entry into our study, this patient was treated with mitoxantrone and interferons, and further along the disease course, natalizumab therapy had to be established due to ongoing high disease activity (patient 1, Table 1; and Fig 2).

Secondly, a similar female RRMS patient (Table 1, patient No 7) also with a very active disease course 5 years in duration, showed similar values for RNFL (right eye, global, 110 μm; left eye, global, 107 μm) and for TMV (right eye, 8.87mm³ and left eye, 8.87 mm³) as the patient described above (Table 1, patient 1). It should be noted that patient 7 is 7.5 years older than patient 1, but had nearly the same lesion load in her visual pathway as patient 1, with (1) an absolute concentration of NAA in her NAWM (11.97 mM) and a ratio of NAA to Cr (1.98) within normal range, and no other signs of brain atrophy. Patient 7 had an EDSS of 4.0. This patient also had to be treated with natalizumab later in the disease course due to high disease activity (Table 1).

Thirdly, a female RRMS patient with previous ON of her right eye (patient No 20, Table 1), a rather long and active disease course (5.0 years), but otherwise with normal findings for absolute concentration of NAA in NAWM (13.85 mM), a ratio of NAA to Cr of 2.07 and no signs of brain atrophy with normal brain atrophy indices and the lowest visual pathway lesion load of all 28 included MS patients, had a significantly reduced RNFL on her right eye compared to her left eye (global, 87 μm vs. 100 μm; T, 67 μm vs. 86 μm; TS, 148 μm vs. 165 μm; TI, 110 μm vs. 159 μm; I, 100 μm vs. 125 μm). Interestingly, the TMV was within normal range and showed no side difference (right eye, 8.44 mm³ vs. left eye, 8.49 mm³). Why the RNFL reduction in the temporal sectors of the patient’s right eye was not associated with the TMV in this patient remains unclear. Her EDSS was 1.5.

Fourthly, a male SPMS patient (patient 26, Table 1) with a very long disease duration (25 years) and long periods of highly active disease course with prior ON of each eye had unexpectedly normal values for RNFL (global: right eye, 111 μm; left eye 103 μm) and TMV (right eye, 8.1 mm³; left eye, 7.95 mm³). This seems even more paradoxical as: (1) brain atrophy was obvious, (2) the brain atrophy indices were partly out of range (maximum width of 3^rd^ and 4^th^ ventricles, 8.9 mm and 12.9 mm respectively; MFSS 3.0; MIF 2.0; MSF 2.8), (3) NAA levels in the NAWM were markedly reduced (absolute concentration, 8.25 μm), and as (4) the lesion load of the visual pathway was nearly the highest of all included patients (2^nd^ rank). Of note is that the RNFL on both sides was within ‘normal range’, but significantly reduced only in his left eye compared to the fellow eye, although he experienced ON in both eyes. In any case, the RNFL and TMV were found to be reduced compared to the patient’s right eye. (Reliable normative databases do not exist for RNFL or TMV, and the individual baseline levels before disease onset is of course unknown). His EDSS was 6.5.

Finally, and in clear contrast to the last patient described, the fifth (male SPMS) patient (patient 24, Table 1) did not experience previous ON but was of similar age as patient 4 at entry to the study (46.5 years). While the total disease duration was significantly less (6.5 years vs. 25 years), disease activity and disability was comparable high (EDSS 6.5). Brain atrophy was less obvious, the brain atrophy indices were only partly out of range, but less so compared to the last patient (maximum width of 3^rd^ and 4^th^ ventricles, 8.7 mm and 10.6 mm; MSF 2.0). The lesion load in the visual pathways was a third less, while the brain metabolites were within normal range (NAA, absolute concentration, 10.59 mM; ratio of NAA to Cr, 1.84). However, RNFL values were significantly reduced (RNFL global, 81 μm and 78 μm). The TMV correlated well to RNFL values (right eye, 8.01 mm³ and left eye, 7.33mm³).

In summary, we found no strict correlation between the specific parameters examined (Fig 1, Table 6). The RNFL thickness of patients’ eyes with ON compared to their unaffected fellow eyes, to healthy controls and the internal databases of OCT devices has been found reduced in most, but not all patients [33]. The set of various parameters was complex and heterogeneous, and did not strictly follow a unique distinct pattern, which would have served for further forecast analysis (and prediction).

## Discussion

It has long been recognised that neither lesion load nor brain- or spinal cord atrophy correlates strictly to the degree of disability in MS. MS patients with high relapse rates and high lesion load, but only mild impairment are characteristic of the well-known ‘clinico-radiological paradox’ [3; 43]. The cause and mechanisms for this remain to be elucidated. Furthermore, the cause of retinal measurements (RNFL, TMV etc.) that are in contrast to clinical symptoms and MRI monitoring, is the subject of ongoing debate since the OCT was introduced for ‘monitoring neurodegeneration of MS patients’ since 2006, and even more so since OCT was proposed as a surrogate for MRI monitoring.

This replacement of MRI with OCT is remarkable as only few studies with consistent and comparable study design and methodology have yet been published. Most of the studies (search terms, “OCT”, “MS” and “brain atrophy”, see above, introduction [6–8; 16; 20–22; 25]) were performed, (1) with conventional time domain OCT [6–8; 16; 21–22], and (2) the case numbers of the studies were generally low, even when they were published in high-ranked journals (ref. 6 [6], 11 patients with probable or clinically-definitive MS according to Poser’s criteria; ref. 7 [7], 20 RRMS, 15 SPMS, and 5 PPMS patients; ref. 8 [8], naïve RRMS patients with disease duration up to 20 years; ref. 9 [9], 56 CIS patients, two-thirds with dissemination in space according to the Barkhof’s criteria and one-third with previous unilateral ON; ref. 16 [16], 104 RRMS patients; ref. 20 [20], 63 RRMS patients; ref 21 [21], 10 CIS and 34 RRMS; ref. 22 [22], 29 benign MS patients, i.e. EDSS≤3.0, disease duration ≥ 15 years; ref. 25 [25], 68 RRMS and 9 SPMS).

According to Popper’s ‘falsifiability’ theorem, studies with low case numbers allow the confirmation or rejection of a general hypothesis, and this is suggested to be valid in the case of MS patients. In other words, accurately-derived negative results need not be compensated by a high number of cases: ‘one negative result may discard a theory until the opposite is proven’, particularly, as the situation in healthy controls of different sex, background and age is not clear. Large normative databases for RNFL and TMV for the different OCT devices are still not available. More importantly, daily physiological variations of the retina might significantly exceed the anticipated annual change of RNFL thickness (0.1–2 μm) [32]. Balk et al. identified hydration status as a crucial confounder for any retinal scan, statistical analysis and further interpretation, even in young healthy controls. Retinal changes were found to be significantly reduced between baseline and first follow-up scans performed before and after a 10km charity-run (dehydration status). To rehydrate, the participants were advised to drink water and sports drinks. Significant RNFL and TMV reduction were found to be fully reversible in the follow-up scans only 1 to 1.5 hours after the 10km charity run. It is generally accepted to refer to this phenomenon as ‘RNFL reduction’ rather than ‘RNFL thinning’ [32]. Of note, Balk et al relativized this hydration-/rehydration-effect to <1% of the OCT data variation in a recently published study [44] when they reinvestigated the 26 healthy test persons of their original study [32]. Balk et al interpreted their new data as follows,”*(1) Normal variation of OCT data may mask small degrees of neurodegeneration*, *(2) Hydration related cellular volume changes may be a cause for OCT data variation*, *(3) their prospective trial demonstrates that hydration causes < 1% of OCT data variation and (4) Trials using OCT will need to consider normal variation*.” [44]. Balk et al opened very interesting, new questions such as variations of the macular RNFL, ganglion cell layer, inner plexiform layer, inner nuclear layer, outer plexiform layer and outer nuclear layer, and variations caused by instrument/software related factors [44], which may explain the RNFL variations found in their first study apart from a hydration/rehydration-effect only [32]. It is essential that the study protocol in their second study was changed: they excluded a dehydration of participants, hence only the effects of hydration on the retina were studied, and may explain the partly controversial results of their both studies [32; 44]. To the best of our knowledge, it has yet not been studied in detail and in a large cohort of MS patients if hydration related cellular volume changes may be a cause for OCT data (and/or MRT?) variation. However, it is well known that many MS patients limit their fluid intake to reduce their bladder urge during their daily routine [45].

If current investigations (OCT, MRI or MRS) are still too imprecise to detect and monitor the subtle, early changes that exist in MS patients (or that cannot be separated from gross ON changes), it should at least be possible to detect the hypothesized changes in all remaining MS patients with high activity, long disease courses and obvious progression (progressive relapsing MS, SPMS and PPMS). According to this hypothesis which is referred to by several studies, many patients would indeed escape detection in very early disease stages or if the disease follows a benign course [6–8; 16; 20–22; 25]. The OCT data published to date indicate to a new paradox: ‘CROP–the clinico-radiologico-ophthalmological paradox’.

The aim of our detailed study was to confirm or discard CROP. The power of our study was that: (1) MRI/MRS and OCT were performed on the same day immediately after each other, (2) that the MS patients were encouraged to drink sufficiently before the examinations [32], (3) that advanced high-resolution SD-OCT technique and (4) MRI/MRS technique was used on (5) well classified MS patients with partly high disease activity and/or long disease courses, and finally, (6) that all data are traceable to allow readers their own interpretation [36–37; 39]. Most importantly, the disease activity remained high in further follow-up appointments with a median observation period of 22 ± 0.5 months, but showed neither significant reduction of the RNFL nor the TMV [36–37; 39]. This is of greater interest as we balanced our ‘inclusion criteria’ to ‘false negative’ rather than ‘false positive’ inclusion criteria; in other words, we included only patients that would be classified as ‘MS patients’ no matter which diagnostic criteria would have been used.

However, we could not identify a strict correlation or rule that applied to all MS patients which allows a reliable prediction either of RNFL/TMV reduction over time, other examined parameters or disease course. This might be explained by: (1) MS patients that follow a benign course, (2) relapsing-remitting courses without any disease progression (and pathological changes) between relapses [3–4; 43], (3) focal lesions in the spinal cord or certain regions in the brain such as the brain stem or cerebellum etc. which correlate better to physical impairment and hence, the EDSS, (4) methodological limitations that make it impossible to detect all ongoing, subtle changes under the detection limits (less than ±2–4μm RNFL) of available SD-OCT devices [4–5; 25–27], (5) subtle changes that cannot be separated from gross pathological changes by focal lesions in the retina or anterior visual pathway [20; 25–27], or [6] that other, as of yet undefined pathogenetic mechanisms of degeneration and repair affect the CNS tissue differently (e.g. [45–48]).

We conclude that the well-known ‘clinico-radiological paradox’ or ‘radiological isolated syndrome’ [3–4; 43] also applies to the OCT, i.e. that every patient seems to follow his own individual course with an individual set of para-clinical parameters. As others we think that it is to premature to suggest OCT as surrogate marker or tool to measure and monitor cerebral and spinal atrophy [49–50].
